# High-Affinity α-Conotoxin PnIA Analogs Designed on the Basis of the Protein Surface Topography Method

**DOI:** 10.1038/srep36848

**Published:** 2016-11-14

**Authors:** Igor E. Kasheverov, Anton O. Chugunov, Denis S. Kudryavtsev, Igor A. Ivanov, Maxim N. Zhmak, Irina V. Shelukhina, Ekaterina N. Spirova, Valentin M. Tabakmakher, Elena A. Zelepuga, Roman G. Efremov, Victor I. Tsetlin

**Affiliations:** 1 Shemyakin-Ovchinnikov Institute of Bioorganic Chemistry, Russian Academy of Sciences, 16/10 Miklukho-Maklaya Street, 117997 Moscow, Russia; 2 School of Biomedicine, Far Eastern Federal University, Bldg. M, 10, Ajax Village, Russky Island, Vladivostok 690000, Russia; 3 G.B. Elyakov Pacific Institute of Bioorganic Chemistry, Far Eastern Branch, Russian Academy of Sciences, 159, Prospect 100 let Vladivostoku, 690022 Vladivostok, Russia; 4 National Research University Higher School of Economics, Myasnitskaya ul.20, 101000 Moscow, Russia

## Abstract

Despite some success for small molecules, elucidating structure–function relationships for biologically active peptides — the ligands for various targets in the organism — remains a great challenge and calls for the development of novel approaches. Some of us recently proposed the Protein Surface Topography (PST) approach, which benefits from a simplified representation of biomolecules’ surface as projection maps, which enables the exposure of the structure–function dependencies. Here, we use PST to uncover the “activity pattern” in α-conotoxins — neuroactive peptides that effectively target nicotinic acetylcholine receptors (nAChRs). PST was applied in order to design several variants of the α-conotoxin PnIA, which were synthesized and thoroughly studied. Among the best was PnIA[R9, L10], which exhibits nanomolar affinity for the α7 nAChR, selectivity and a slow wash-out from this target. Importantly, these mutations could hardly be delineated by “standard” structure-based drug design. The proposed combination of PST with a set of experiments proved very efficient for the rational construction of new bioactive molecules.

The general concept of structure–function dependencies is one of the core principles in natural sciences. It underlies the intriguing possibility to rationally design biological molecules that would possess the desired characteristics required for novel research instruments or medicines. Although long proclaimed, the rational design of biological molecules (e.g., drug design) is still more art and fortune than well-established technology. This is especially true when, rather than talking about small molecules (where drug design strategies have achieved considerable success^1^,^2^,^3^,^4^,^5^,^6^,^7^), we examine bioactive peptides, which are versatile bioregulators and target many receptors and ion channels in the organism^8^,^9^,^10^,^11^,^12^, most significantly in the nervous system. Because protein–ligand binding critically depends on the spatial distribution of a number of physical properties over the interacting surfaces, a detailed characterization of the latter is indispensable in order to understand the interaction mechanisms. Being complex and delicate even in the case of small ligands, the surface organization of relatively large and highly flexible bioactive peptides is much more difficult to explore, thus calling for new ideas and solutions.

Recently, some of us have proposed the Protein Surface Topography (PST) approach^13^, which extends the arsenal of computational structural biology by offering a method that considers bioactive peptides and their targets as interacting surfaces. These surfaces are subjected to simplification and transformation into a machine-tractable format of regular projection maps, which mirror biomolecules’ properties and enable group analysis, yielding a pattern that defines activity/selectivity for a group of molecules. This approach was initially tested on a set of neurotoxic peptides from scorpions’ venoms^14^. In the present work, we apply PST to a set of α-conotoxins, small neurotoxic peptides from the venoms of predatory marine *Conus* sp. snails, since they are very effective and often quite selective antagonists targeting distinct subtypes of nicotinic acetylcholine receptors (nAChRs) in the nervous system and other tissues (see reviews^15^,^16^,^17^). α-Conotoxins are useful tools in nAChR research and look promising for the design of new drugs because of the involvement of nAChRs in a number of pathologies (Parkinson’s and Alzheimer’s diseases, schizophrenia, myasthenia, nicotine addiction)^18^.

α-Conotoxins belong to neurotoxins, a class of biomolecules that have earlier played an important role in the identification and purification of the nervous system’s receptors. For example, the low-molecular weight antagonist strychnine was used to isolate the glycine receptor^19^, and snake venom α-neurotoxins (for example, α-cobratoxin, αCtx) were employed for affinity purification of the muscle-type nAChR from a *Torpedo* electric ray^20^ and for the nAChR from a rat brain^21^, later identified as the neuronal α7 nAChR^22^; the radioiodinated α-bungarotoxin ([^125^I]-αBgt) was used to determine the receptor’s binding parameters. However, it is not only history — both mentioned neurotoxins, along with α-conotoxins and some other compounds, are routinely applied in pharmacology and also as accurate tools to probe the spatial structure of the relevant receptors. For example, the d-tubocurarine’s mimetics are widely used as peripheral myorelaxants^23^, while the X-ray structures of the complexes formed by acetylcholine-binding proteins (AChBPs, excellent models for the ligand-binding domains of all nAChRs and other Cys-loop receptors^24^) with αCtx^25^, strychnine and d-tubocurarine^26^ provided valuable information about the binding sites in the nAChRs.

α-Conotoxins are excellent tools for research on nAChRs and have a certain advantage over snake venom α-neurotoxins, which also target these receptors. Traditionally, the so-called short chain α-neurotoxins from snake venom were used as markers of the muscle-type nAChRs, while the long-type α-neurotoxins αBgt and αCtx with a similarly high affinity inhibit muscle and two neuronal subtypes, namely α7- and α9-nAChRs (see reviews^12^,^27^). On the other hand, α-conotoxins provide better possibilities for distinguishing between various subtypes of neuronal nAChRs, differing not only in subunit types, but also in their stoichiometry (see reviews^28^,^29^). In spite of the large number of α-conotoxins that have been isolated from venoms, synthesized as their analogs or based on sequences identified in transcriptomes, designing potent and selective ligands for a particular nAChR subtype is still a challenge. Of special interest are α7 nAChRs, since they are involved in diverse physiological functions and impairments in their activity are associated with many diseases, including psychiatric and neurodegenerative (see reviews^30^,^31^,^32^).

Here, the PST approach^13^ guided the computational design, synthesis and multifunctional testing of three novel α-conotoxin analogs targeting α7 nAChRs with high affinity. The results obtained show that PST-guided design combined with the proposed advanced testing protocol represent a promising tool for the discovery of the structure–function relationships of bioactive peptides and the design of more potent ligands.

## Results

### Protein surface topography provides a rational framework for the design of peptides with increased activity

**Figure 1:**
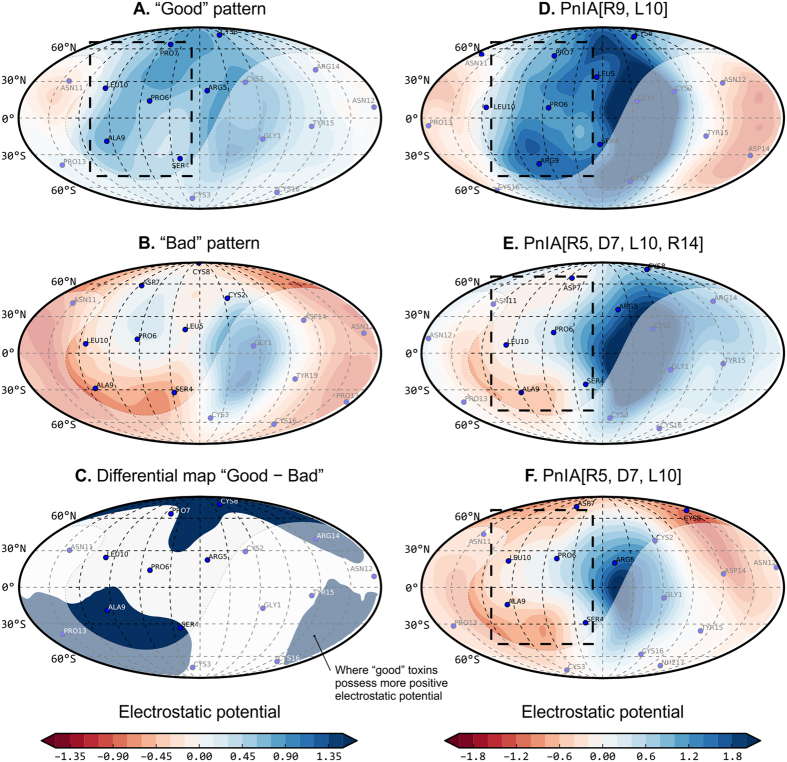
Design of potent α7 nAChR inhibitors on the basis of α-conotoxin PnIA. All panels (except C) are the spherical projection maps of surface electrostatic potential (ELP) for a conotoxin or conotoxins group, produced with the Protein Surface Topography approach. These maps depict the conotoxins’ whole surface; the area that should be exposed to solvent in the PnIA–AChBP complex is *filled with semi-transparent color*. However, one should bear in mind that binding to the nAChR may differ. (**A**) Pattern of “good” toxins. (**B**) Pattern of “bad” toxins. These two panels are colored by ELP according to the scale on the *bottom left*. (**C**) Differential map “good–bad”. The blue color denotes areas where ELP is more positive in “good” toxins. (**D**) Map for PnIA[R9, L10], which was designed, synthesized and tested in this work. (**E**,**F**) Maps for PnIA[R5, D7, L10, R14] and PnIA[R5, D7, L10], respectively, which were synthesized and tested previously^35^, and do not exhibit high inhibiting potency. Panels D–F are colored by ELP according to the scale on the *bottom right*. The area of maximum difference between our current best blocker and two other toxins is denoted with a *dashed black box*; it encloses residue 9, where substitution to Arg is shown to be important (this work). The figure was prepared with our in-house Protein Surface Topography software^13^, which is currently available only on request.

To date, there is a considerable body of results on α-conotoxins blocking the α7 nAChR. These data are a firm basis for structure–function analysis aiming to map the ligand pharmacophores and to choose substitutions that would modify their activity in a desirable way. In this work, our aim was to design a novel α7 nAChR ligand with enhanced affinity. The computational strategy comprised several steps (summarized in Fig. S1):

1. Establishing a database of α-conotoxins with known α7 nAChR blocking activity and ascribing them to three groups: “good”, “average” and “bad” (see *Methods*).
2. Creating a structural database: 3D models of all relevant α-conotoxins were either obtained from PDB or built by homology.
3. Calculating the molecular dynamics (MD) of the toxins in a water box.
4. Building MD-averaged 2D spherical maps (PST-maps or “globes”) of hydrophobic and electrostatic properties distributed over the toxins’ entire surface by employing the Protein Surface Topography approach (for explanation, see Fig. S1 and ref. 13).
5. Analyzing relationships between the toxins’ activity and properties, visualized in 2D spherical maps. Constructing group-averaged maps for “good” and “bad” toxins (Fig. 1A,B, respectively).
6. Constructing a differential map emphasizing the most prominent differences between the groups of “good” and “bad” toxins (Fig. 1C).
7. Designing mutant toxins whose PST-maps fit those obtained for the “good” toxins by repeating steps 3–4 above.

This analysis revealed that “good” toxins appear to have more positive electrostatic potential: comparing panels A (which is predominantly blue) and B in Fig. 1, a regularity can be seen in the middle row of Fig. S1. The differential map “A–B” (Fig. 1C) reveals the presence of positive electrostatic potential in the “south-west” area of the toxin’s map (“globe”), which is consistent with Arg9 residue that resides in this position in many “good” toxins — such as ArIB and its mutants — but not in PnIA or any of its mutants. This observation, supplemented with an abundance of characterized PnIA analogs (a fact that significantly increases the reliability of the PST application) prompted us to obtain a series of novel PnIA analogs with А9R substitution. Another potentially favorable substitution is A10L, which has already been shown to increase PnIA affinity for the α7 nAChR^33^,^34^. The additional two substitutions that can probably “improve” the electrostatic map of PnIA and make it more consistent with the average “portrait” of a “good” toxin are arginine residues in positions 5 and 14, both of which have previously been reported to increase mutants’ activity as well. At the same time, it is not only the positive charge itself that matters, but also its peculiar distribution over the toxin’s surface. For example, several previously known mutants with a net positive charge, e.g. PnIA[P7R, A10L] (charge +1) or PnIA[L5Y, P6R, P7R, A10L, D14R, Y15W] (charge +4) have large spots of positive electrostatic potential on their surfaces (Fig. S7) but moderate or low activities (see Table S1) — this is due to a suboptimal distribution of potential, which is easily tracked and analyzed with PST.

Finally, we synthesized three α-conotoxin PnIA analogs: PnIA[R9], PnIA[R9, L10] (both with a charge of +1), and PnIA[R5, R9, L10, R14] (charge of +3). These substitutions were not arbitrarily chosen: PST-analysis allows us to determine our most potent variant (see below; PnIA[R9, L10]) along with two close moderately-active analogs that were studied previously (PnIA[R5, D7, L10, R14] and PnIA[R5, D7, L10])^35^. A comparison of electrostatic maps reveals a closer resemblance of the most potent variant to the averaged “good portrait” of an α7 blocker (comparing panels A and D in Fig. 1), while the “moderate analogs” (panels E and F) more closely resemble the averaged “bad portrait” (panel B in Fig. 1).

### Designed PnIA analogs are high-affinity α7 nAChR blockers

**Figure 2:**
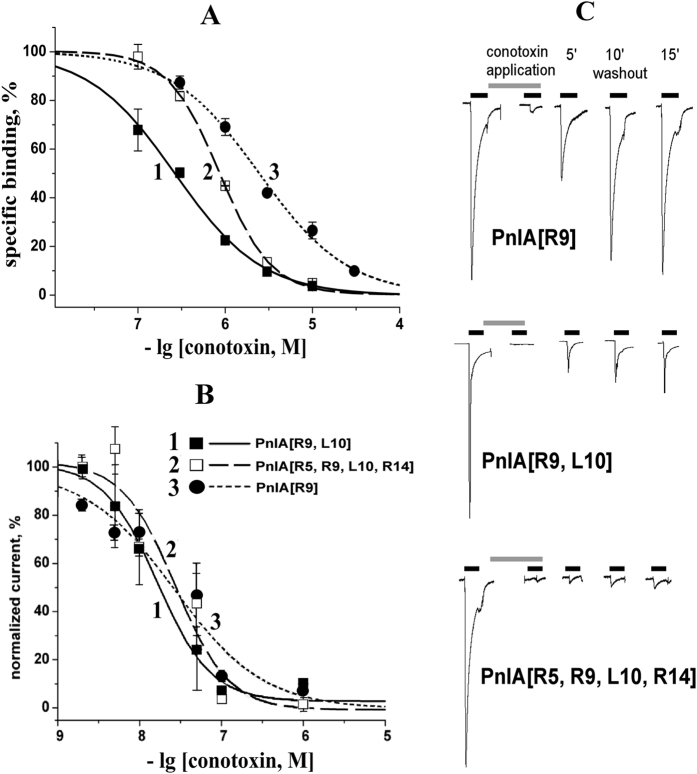
Designed α-conotoxin PnIA analogs are potent α7 nAChR inhibitors. (**A**) Radioligand competitive binding analysis: inhibition of [^125^I]-labeled αBgt binding to the α7 nAChR transfected in GH_4_C_1_ cells with three PnIA analogs. Each point is the mean ± s.e.m. value of two measurements for each concentration in two independent experiments. The curves were calculated from the means ± s.e.m. using the ORIGIN 7.5 program (see *Methods*). The respective IC_50_ values (mean ± s.e.m.) and Hill coefficients are provided in Table 1. (**B**) Electrophysiology studies of the same molecules on the α7 nAChR expressed in *Xenopus laevis* oocytes (mean ± s.e.m., *n* = 3–4). The respective IC_50_ values are provided in Table 1. (**C**) Raw recordings of electrophysiology currents reveal essentially different blocking potency and washout rates for each molecule.

The activities of novel α-conotoxin PnIA analogs were assayed by three independent methods: radioligand analysis, electrophysiology and calcium imaging. In a competitive radioligand assay with [^125^I]-labeled αBgt, all three synthesized analogs completely (but with varying affinities) displaced the radioligand from the α7 nAChR transfected in GH_4_C_1_ cells (Fig. 2A). A similarly potent inhibition was also observed for acetylcholine-binding proteins from *Lymnaea stagnalis* and *Aplysia californica* (*Ls*- and *Ac*-AChBPs), which are excellent spatial homologs of the ligand-binding domain of the α7 nAChR and are often used in parallel with full-size nAChRs. The calculated binding parameters derived from two independent experiments performed for each analog and each target are provided in Table 1. According to the competitive radioligand assay, the most potent α7 nAChR inhibitor was the PnIA[R9, L10] analog with IC_50_ = 270 ± 10 nM and a Hill coefficient of 0.87 ± 0.03. Among the PnIA analogs, it has the best affinity towards this receptor subtype, evaluated by competition with αBgt.

Earlier, we revealed that the application of the [^125^I]-labeled αBgt (which binds to the α7 nAChR almost irreversibly) as a radioligand to evaluate the binding parameters of α-conotoxins (rapidly washing out from the complex with the receptor) often results in unreasonably high IC_50_ values^35^. For this reason, all three new analogs were assayed for their affinities towards the α7 nAChR, expressed in *Xenopus laevis* oocytes in electrophysiology studies by their ability to block acetylcholine-induced currents through the receptor. Two-electrode voltage clamp experiments demonstrated high-affinity blocking of α7 nAChR currents by all three tested analogs (see Fig. 2B for dose-response curves). The calculated IC_50_ values for PnIA[R9], PnIA[R9, L10] and PnIA[R5, R9, L10, R14] were all around 20 nM (see Table 1 for details) which are at least 10-fold different from the values evaluated in competition with the [^125^I]-labeled αBgt (see *Discussion*).

Apart from blocking potency, there is another important pharmaceutical characteristic: the stability of the complex, represented by the apparent washout rate. This characteristic is considerably different for the obtained PnIA analogs (Fig. 2C): the PnIA[R9] was completely washed out from the complex with the α7 nAChR in 15 min, while only half of the bound PnIA[R9, L10] was washed out in this time interval. Moreover, the PnIA[R5, R9, L10, R14] was found to be irreversibly bound to the α7 nAChR during a 15 min washing. This feature (together with their nanomolar affinities for the α7 nAChR) makes the two last analogs similar to snake venom long-type α-neurotoxins (such as αBgt or αCtx).

Therefore, we decided to check whether the PnIA[R5, R9, L10, R14] analog, having the slowest wash-out rate, had acquired the ability to interact effectively with the muscle-type nAChR as is the case with αBgt or αCtx. This was done via the calcium imaging method using the mouse muscle α_1_β_1_δε-nAChR, heterologously expressed in the neuroblastoma Neuro2a cell line (see *Methods*). It was shown that the PnIA[R5, R9, L10, R14] at a concentration of 0.55 μM does not exert any reliable effect on the acetylcholine-induced rise in [Ca^2+^]_i_ (Fig. S2). This demonstrates that PnIA[R5, R9, L10, R14] does not interact with muscle-type receptors, suggesting higher selectivity towards the α7 nAChR compared with that of αBgt or αCtx.

### Preparation of [^125^I]-labeled derivatives of α-conotoxin PnIA analogs and detection of their probable allosteric binding site on the α7 nAChR

**Figure 3:**
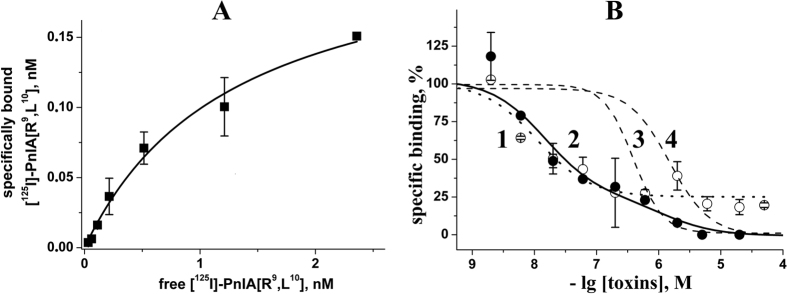
(**A**) Saturation binding curve for [^125^I]-PnIA[R9, L10] (from 0.05 to 2.5 nM) interaction with the α7 nAChR transfected to GH_4_C_1_ cells. The respective K_d_ and B_max_ values (mean ± s.e.m.) calculated from a single experiment (duplicated for each point) were 1.30 ± 0.28 nM and 0.23 ± 0.02 nM. (**B**) Inhibition of [^125^I]-PnIA[R9, L10] binding to the same receptor: (1) α-cobratoxin (open circles, dotted line), (2) α-conotoxin PnIA[R9, L10] analog (filled circles, solid line), (3) α-conotoxin PnIA[R5, R9, L10, R14] analog (dashed line) and (4) α-conotoxin PnIA[R9] analog (dashed line). To simplify the figure, the data points for the last two analogs were omitted, and only the calculated inhibition curves are shown. Each point is the mean ± s.e.m. value of two measurements for each concentration in two independent experiments for (3) and (4) and in three independent experiments for (1) and (2). The curves were calculated from the means ± s.e.m. using the ORIGIN 7.5 program (see *Methods*). The IC_50_ value (mean ± s.e.m.) for the α-cobratoxin was 11 ± 3 nM from 75% binding sites, and the respective values of complete inhibition for α-conotoxins are provided in Table 1.

To evaluate the possibility of using the new PnIA analogs as tools for studying α7 nAChRs, radioactive forms of all three peptides were prepared. Mono-[^125^I]-labeled derivatives ([^125^I]-PnIA[R9], [^125^I]-PnIA[R9, L10] and [^125^I]-PnIA[R5, R9, L10, R14]) with specific radioactivity of 2000 Ci/mmol (see *Methods* and Fig. S3) were used in the radioligand assay. The [^125^I]-PnIA[R5, R9, L10, R14] demonstrated an excessive level of nonspecific binding, thus hampering its application as a reliable radioligand. Therefore, most studies were carried out with [^125^I]-PnIA[R9, L10] (its non-radioactive version also showed a slow washout rate from the α7 nAChR — see Fig. 2C). Saturating GH_4_C_1_ cells by the [^125^I]-PnIA[R9, L10] transfected with the α7 nAChR (Fig. 3A) allowed us to calculate very reliable binding parameters: *K*_d_ = 1.30 ± 0.28 nM and B_max_ = 0.23 ± 0.02 nM.

However, the kinetics of [^125^I]-PnIA[R9, L10] washout from the receptor revealed an interesting effect. Adding excess concentrations of αCtx (the classic competitive antagonist for the α7 nAChR) could not completely remove the radioligand from the target even after a 2-hour incubation, thus suggesting an additional (allosteric) binding site besides the orthosteric one for agonists and competitive antagonists such as αCtx (Fig. S4). The portion of these additional sites in the total [^125^I]-PnIA[R9, L10] binding to the α7 nAChR was estimated from these data to be 25–30%.

We studied the dose-response inhibition of the [^125^I]-PnIA[R9, L10] binding to the α7 nAChR by αCtx and all three α-conotoxin PnIA analogs. We found that αCtx up to a 50 μM concentration does not displace ≈25% of the radioligand (Fig. 3B). The calculated IC_50_ value for “replaceable” binding at the orthosteric site was 11 ± 3 nM and its Hill coefficient was 1.0 ± 0.3.

On the other hand, the displacement of the [^125^I]-PnIA[R9, L10] by the same non-radioactive analog constitutes 100% (Fig. 3B). The calculation of the data obtained with the OriginPro 7.5 program revealed the clear two-site nature of the binding inhibition: the IC_50_ value for the high-affinity site was 15 ± 6 nM (see Table 1), which practically coincides with that for αCtx (11 ± 3 nM), whereas the affinity for the low-affinity (“allosteric”) binding site of the radioligand was about 1 μM.

It is worth mentioning that two other α-conotoxin PnIA analogs also completely (although with lower affinity) displaced the [^125^I]-PnIA[R9, L10] from the α7 nAChR (Fig. 3B). The calculated IC_50_ values were very close to the affinities obtained for these analogs in competition with the [^125^I]-labeled αBgt in binding to this receptor (see Table 1).

The possibility of the existence of such an additional “allosteric” binding site on the α7 nAChR was even more clearly demonstrated with the [^125^I]-PnIA[R5, R9, L10, R14] analog in competition with different concentrations of its non-radioactive form and αCtx (Fig. S5). The complete displacement by the first one (IC_50_ = 1.6 ± 0.4 μM) obviously contrasts with the poor competitiveness of αCtx, which even at concentrations of up to 100 μM failed to achieve a 50% inhibition. These results can be explained by a higher proportion of “allosteric” binding sites for the PnIA[R5, R9, L10, R14] on the α7 nAChR and by the high potency of the interaction, in agreement with a slow washout rate of this analog from the receptor detected in electrophysiological testing (see Fig. 2C).

## Discussion

The rapid progress in the elucidation of the structure of Cys-loop receptors that we see today started with X-ray diffraction and electron microscopy of the nAChR from the electric organ of the *Torpedo marmorata* at the end of the 1970-s^36^,^37^. Nevertheless, for the whole large family of muscle-type and neuronal nAChRs, the cryo-electron microscopy structure of this receptor with a resolution of about 4 Å published by Nigel Unwin about a decade ago^38^ and the more recent structure describing acetylcholine-induced changes^39^ are the only ones available.

As mentioned in the *Introduction*, several structures for snake venom neurotoxins complexes with nAChR models are known, namely acetylcholine binding proteins (AChBPs)^25^, with ligand-binding domains of α_1_ and α_9_ subunits^40^,^41^ and a chimera of the α7 subunit and AChBP^42^. In the case of α-conotoxins, the only published X-ray structures of complexes are for *Aplysia californica* AChBP^43^,^44^,^45^,^46^,^47^,^48^, which are used to infer information about the binding sites within the nAChRs. However, the available computational methods used for this are far from ideal: the reliability of the predicted structures of the nAChR complexes is not particularly solid.

For this reason we employed a novel computational approach in this work — PST^13^, which allowed us to observe structure–function relations for bioactive peptides, starting from their structures/models and a set of corresponding measured activities — not requiring support from the spatial structures of receptor complexes. PST proceeds from the organization of the peptide surface as a whole, comprising its dynamic fluctuations and the distribution of physicochemical properties over it. Unlike structure-based drug design, PST is similar to QSAR, albeit without the classical descriptors, but rather a set of “globe maps”. Here, a set of electrostatic “maps” (see Fig. 1) reveal a dynamic amphiphilic “portrait” of PnIA analogs, suitable for the discovery of regularities and structure–function relations. Our analysis had the advantage of covering a large set of data, both our own^35^ and those available in existing literature^16^ on α-conotoxin affinities for AChBPs and the α7 nAChR.

One of the main conclusions of the performed PST analysis is the requirement of Arg9 in the α-conotoxin PnIA necessary to attach it with high affinity to both AChBPs and α7 nAChRs. The experimentally measured binding parameters (Table 1) confirm this conclusion. The most active novel peptide PnIA[R9, L10] was the most potent in competition with the [^125^I]-labeled αBgt compared with the best analogs proposed earlier based on the standard protocols of docking and molecular dynamics^35^: 270 nM *vs*. 670 nM.

Surprisingly, Arg9 (and also Arg5 and Arg14) would not seem a logical substitution from the point of view of “standard” structure-based drug design if we start with the X-ray structure of the PnIA analog bound to *Ac*-AChBP^43^. There is no negatively charged partner (Asp or Glu residues) in the vicinity, although the binding site has overall negative electrostatic potential, apparently created by several acidic residues located “at the edge” of the binding site in both AChBP and the α7 nAChR. Therefore, even the availability of a spatial structure for the related complex does not provide a reliable framework for appropriate design in this case, substantiating the necessity of a ligand-based approach, facilitated in our case by PST.

Conotoxins are not the first application of PST for the analysis of peptides’ structure and function. Some time ago, we used it to analyze a set of so-called α-neurotoxins from scorpion venom that affect the voltage-gated sodium channels of different phyla: insects, mammals, or both. A similar approach helped us discover a “selectivity module” within peptides responsible for targeted action^14^. Moreover, we were able to predict the selectivity of a peptide with unknown parameters and confirm it experimentally.

What is immediately noticeable is the relatively large difference between the binding parameters measured with different methods. In our previous publication for another set of α-conotoxin PnIA analogs^35^, the weakest affinity was found in competition with the radioactive αBgt: most probably because the experiments had to be done within a very short time to measure the decrease of the initial rate of the irreversible αBgt binding. One should also bear in mind that the α7 nAChR has 5 orthosteric binding sites for agonists/competitive antagonists, and that radioligand analysis monitors the displacement of the αBgt from all of them. On the contrary, to block the α7 nAChR function in electrophysiology tests, one molecule of the antagonist is sufficient^49^. In accordance with earlier findings^35^, the binding parameters obtained in the present communication from electrophysiology experiments on the α7 nAChR expressed in *Xenopus* oocytes (Fig. 2B) are 1–2 orders better than those measured by competition with the αBgt in the radioligand assay (Table 1).

Clearly, electrophysiology can be recommended for an accurate assessment of the activity of novel α-conotoxins, but radioligand analysis should not be neglected either. It is clear that competition with the radioactive αBgt indicates binding in the “classical” binding sites for agonists and competitive antagonists, while the inhibition of ion currents for the α7 nAChR expressed in *Xenopus* oocytes or cell lines does not provide information about the character of the binding sites. In fact, a combination of binding studies (using either radioactive or fluorescent ligands) and testing of functional activities may shed light on the mechanisms of binding: for example, using αCtx, its fluorescent derivative and the joint application of electrophysiology, calcium imaging and fluorescence spectroscopy, it was discovered that αCtx inhibits the GABA-A receptors by binding both in the orthosteric and allosteric sites^50^.

To summarize, the novel method of protein surface topography (PST) proposed earlier on purely theoretical grounds in the attempt to understand the recognition and interaction of proteins by analyzing their exposed surfaces, has been verified in the present work using abundant data on the affinity of α-conotoxins for AChBP and α7 nAChRs. The correctness of the chosen amino-acid substitutions in the synthesized novel analogs of the α-conotoxin PnIA has been confirmed by analyzing their activities using a combination of different experimental approaches: radioligand analysis, two-voltage clamp electrophysiology and fluorescent imaging. It is notable that the “success story” with the new highly active PnIA analogs became possible due to a combined approach using complementary *in silico* and experimental methods. This allowed us to take into account the pros and cons of the approaches and correctly interpret the binding parameters provided by each of these methods. The validity of this first PST-guided design is evident since it provided novel α-conotoxin PnIA analogs with very high affinity for AChBP or the α7 nAChR.

It was specifically the combination of the above-mentioned experimental approaches that allowed us to distinguish those new analogs that were washed out very slowly from the tagged nAChR. This is a very valuable property because, in spite of their reasonably high affinity and selectivity to a distinct nAChR subtype, the fast off rates of α-conotoxins usually preclude their application for detecting one nAChR subtype or another in tissues. Another advantage of combining PST with an array of experimental approaches was the discovery of an α-conotoxin PnIA analog that binds the α7 nAChR with an affinity comparable to that of the αBgt, but contrary to this α-neurotoxin, does not interact with the muscle-type nAChR. Thus, this α-conotoxin might be useful in distinguishing between the two nAChR subtypes.

What about future possible applications of PST? The closest may be the design of α-conotoxins targeting heteromeric α_4_β_2_-nAChRs, which are among the most abundant nAChRs in the human brain and, like the α7 nAChR, are involved in cognitive processes, and whose malfunction is associated with many diseases. For other Cys-loop receptors, information about bioactive peptides that target them is scarce. However, the GABA-A receptor should be mentioned here: a decade ago, it was found that one subtype could bind the αBgt^51^ and it was recently shown that this receptor also binds the αCtx^50^ and some other snake venom neurotoxins^52^,^53^. Moreover, although with a low affinity, the GABA-A receptor can bind the α-conotoxin ImI, which interacts with the α7 nAChR^50^. On the other hand, most endogenous neuropeptides, as well as their close homologs from animal venoms (cf. endothelins and saraphotoxins^54^) target GPCR receptors. Another possibility is the adaptation of the PST approach for small molecule ligands. In view of the abundance of data on binding parameters, available X-ray and NMR structures of these receptors and their complexes, PST may provide new ways to design highly active and selective compounds as potential new drugs.

## Methods

### Database of conotoxins that act on the α7 nAChRs

To perform the structure–function analysis, we established a database of 4/7 α-conotoxins and their mutants that inhibit the α7 nAChRs (Table S1). According to this inhibiting activity (IC_50_), the ligands were divided into three groups: “good” (IC_50_ < 16 nM), “average” (39 nM < IC_50_ < 390 nM), and “bad” (IC50 > 390 nM). This database consisted of 39 α-conotoxins collected from literature.

### Molecular dynamics simulations

The discovery of structure–function relationships requires 3D structures of all α-conotoxins, and it will benefit from taking into account their behavior in molecular dynamics (MD) simulations. Several conotoxins’ structures (PnIA, Pdb ID: 1PEN; GID, 1MTQ; Vc1.1, 2H8S; and MII, 1MII) were obtained from PDB, others were modelled using MODELLER 8.2 software^55^ starting from these templates. MD simulations were performed with GROMACS 4.5.2^56^ using Gromos96 45a3 parameters set^57^. The conotoxins’ structures were solvated inside a (3.5–5 nm)^3^ box; a SPC water model^58^ was used; the number of water molecules was 1600–3600. Counterions (Na^+^ or Cl^−^) were added to maintain electroneutrality (0–4 ions). Simulations were carried out with a time step of 2 fs, imposing 3D periodic boundary conditions, in the isothermal-isobaric (NPT) ensemble using a Berendsen barostat^59^ (pressure of 1 bar) and a V-rescale thermostat^60^ (temperature of 37 °C). Van der Waals interactions were truncated using a 1.6 nm spherical cut-off function. Electrostatic interactions were treated with the Reaction-Field algorithm. The length of each MD trajectory was 60 ns, which is enough to sample internal conformational movements for such small and rigid peptides. Figure S6 shows the 3D structure of one of the conotoxins, which is very compact and rigid due to the presence of two disulfide bridges. In addition, stability of the protein conformation is confirmed by the small RMSD values from the starting model and by good preservation of their secondary structure in the course of MD simulations (Figure S6).

### Protein surface topography (PST)

In the PST method^13^, the protein surface is transformed into a sphere, and the sphere-distributed properties, like hydrophobicity in the form of Molecular hydrophobicity potential^61^ (MHP) or electrostatic potential (ELP), are presented as regular spherical projection maps. MHP and ELP were calculated with PLATINUM^62^ and APBS^63^ software, respectively. The regularity of interpolated data allows for simple mathematical operations on the maps, such as summation, subtraction, averaging, etc. Thus, sampling the frames extracted from the MD trajectories (each 100 ps) and spatially superimposing them on the starting structure yields a series of MHP/ELP maps, and averaging results in “dynamic maps” (some of them are provided in Fig. S1) encompassing the dynamic mobility of the toxins’ side chains.

Building group-averaged ELP and MHP maps for “good” and “bad” toxins (Fig. 1A,B), along with the differential map (Fig. 1C), revealed substantial differences and allowed us to rationally introduce several point mutations into the PnIA scaffold that should further increase the affinity of the α7 nAChR.

### Synthesis of a-Conotoxin PnIA Analogs

Solid-phase peptide synthesis was used to prepare all α-conotoxin PnIA analogs as described previously^35^. A preparative purification of the synthesized analogs was carried out on a Gilson HPLC system (333/334 pump with a 215 liquid handler) equipped with an YMC Triart 10 μm (150 × 30 mm) column and a UV detector at 210 and 280 nm. The peptides were eluted in an aqueous gradient of acetonitrile (from 10 to 55% for 30 min) with 0.1% trifluoroacetic acid at a flow rate of 70 mL/min. Chromate-mass-spectrometry analysis was performed using a Thermo Finnigan LCQ Deca XP ion trap instrument with Thermo Accela UPLC system equipped with a Waters Atlantis T3 3 μm (150 × 2 mm) column. Detection was achieved by UV-VIS DAD and full scan MS (ESI+, 150–2000 au). The obtained molecular masses of peptides were very close to the theoretical calculations (Table 2).

### Synthesis of Radioiodinated aBgt and a-Conotoxin PnIA Analogs

αBgt (90 pmoles) dissolved in 20 μL of 125 mM sodium phosphate buffer, рН 7.5, was incubated for 10 min at room temperature with 100 pmoles of Na[^125^I] and a 10-fold molar excess of chloramine T. The reaction products were separated immediately by ion-exchange HPLC in a 5 mM sodium-phosphate buffer, pH 7.5, in a gradient of 0.2 M NaCl 2–62% for 30 min on a column TSKgel CM-5PW (75 × 7.5 mm) at a flow rate of 0.5 mL/min. Detection was carried out at 226 nm and the iodinated products were collected in 0.5 min-fractions. The aliquots of all fractions were counted on a γ-counter Wallac 1470 WIZARD^®^ Gamma Counter (PerkinElmer). Mono- and di-[^125^I]iodinated αBgt derivatives (with approximate specific radioactivity of 2000 and 4000 Ci/mmol) were collected and kept at 4 °C in a 50 mMTris-НС1 buffer, рН 7.5, containing 0.1 mg/ml BSA, for not more than 1 month. We used only mono-[^125^I]iodinated αBgt in our studies.

Before preparing the radiolabeled derivatives of the PnIA[R9], PnIA[R9, L10] and PnIA[R5, R9, L10, R14], we carried out a series of experiments with the nonradioactive [^127^I]-isotope to optimize the reaction and purification conditions, as well as to confirm the structures of iodinated products with MALDI TOF mass spectrometry. The peptides (450 pmoles) dissolved in 20 μL of a 500 mM Tris-HCl buffer, рН 8.0, were incubated for 8 min at room temperature with 500 pmoles of NaI and a 10-fold molar excess of chloramine T. The reaction products were separated by reverse-phase HPLC in an aqueous gradient of acetonitrile (10–50% for 40 min) containing 0.1% trifluoroacetic acid on a column Reprosil-Pur C_18_ AQ (150 × 4.0 mm) at a rate of 0.5 mL/min. The purified peaks (see Fig. S3 for chromatography profile of the PnIA[R9, L10] and PnIA[R5, R9, L10, R14] separation) were analyzed by MALDI TOF mass spectrometry, resulting in the identification of non-modified peptides, mono-[^127^I]-iodinated derivatives and other by-products.

Similar protocols (but with different amounts of reaction components) were applied for the preparation of radioactive derivatives using Na[^125^I] solution. The peptides (85 pmoles) dissolved in 20 μL of a 500 mM Tris-HCl buffer, рН 8.0, were incubated for 15 min at room temperature with 50 pmoles of Na^125^I and a 10-fold molar excess of chloramine T. The reaction products were separated by HPLC under above-mentioned conditions and collected in 0.5 min fractions. The aliquots of all fractions were counted on a γ-counter Wallac 1470 WIZARD^®^ Gamma Counter (PerkinElmer) and mono- [^125^I]iodinated derivatives (with approximate specific radioactivity of 2000 Ci/mmol) of the PnIA[R9], PnIA[R9, L10] and PnIA[R5, R9, L10, R14] were collected. These samples were evaporated (to approx. 50% of their initial volume) to remove acetonitrile and kept at 4 °C in a 50 mM Tris-НС1 buffer with рН 8.0, containing 0.5 mg/ml BSA.

### Radioligand Analysis of a-Conotoxin PnIA Analog Interaction with AChBPs and the a_7_-nAChR

In competition experiments with the [^125^I]-labeled αBgt, all the synthesized α-conotoxin PnIA analogs (the concentration range for each peptide ranged within 0.1–10000 nM) were pre-incubated for 2.5 h at room temperature with *L. stagnalis* or *A. californica* AChBPs (*Ls*- or *Ac*-AChBP) at final concentrations of 2.4 and 140 nM, respectively, or with GH_4_C_1_ cells transfected with human α7 nAChR (final concentrations of 0.4 nM toxin-binding sites) in 50 μL of a 20 mM Tris–HCl buffer, pH 8.0, containing 1 mg/mL of the bovine serum albumin (reaction buffer). After that, the[^125^I]-labeled αBgt was added to the reaction mixtures at a final concentration of 0.2 nM for 5 min. The specific binding was determined by a rapid filtration on double DE-81 filters (Whatman) pre-soaked in the reaction buffer (for AChBPs) or on GF/C filters (Whatman) pre-soaked in 0.25% polyethylenimine (for GH_4_C_1_ cells) and the unbound radioactivity was removed from the filters by washes (3 × 3 mL) with the reaction buffer. Non-specific binding was determined in all cases in the presence of 10 μM α-cobratoxin (2.5 h pre-incubation).

In competition experiments with the [^125^I]-labeled α-conotoxin PnIA analogs (most experiments were carried out with the [^125^I]-PnIA[R9, L10]), the selected α-conotoxin PnIA analogs or α-cobratoxin (the concentration range for the concrete compound ranged within 2-100000 nM) were pre-incubated for 2 h at room temperature with GH_4_C_1_ cells (0.4 nM of toxin-binding sites of α7 nAChR) in 50 μL of a 20 mM Tris–HCl buffer, pH 8.0, containing 1 mg/mL of the bovine serum albumin (reaction buffer). After that, the radioligand (final concentration of 0.2 nM) was added and the reaction mixture was incubated additionally for either 1.5 h (equilibrium binding) or 5 min (initial rate binding) under the same conditions. Non-specific binding was determined in the presence of 20 μM of the respective α-conotoxin PnIA analogs (2 h pre-incubation). The filtration on GF/C filters was performed as mentioned above.

The competition data analyses were fitted using ORIGIN 7.5 (OriginLab Corporation, Northampton, MA, USA) to a one-site dose-response curve with the Equation: % response = 100/(1 + ([toxin]/IC_50_)^n^), where IC_50_ is the concentration at which 50% of the sites are inhibited and n is the Hill coefficient.

The equilibrium saturation binding of the [^125^I]-labeled α-conotoxin PnIA analogs with the α7 nAChR transfected in the GH_4_C_1_ cell line was carried out in 50 μL of a 20 mM Tris–HCl buffer, pH 8.0, containing 1 mg/mL of the bovine serum albumin (reaction buffer) at room temperature. Various concentrations of the radioligand (0.02–2.5 nM) were incubated with the cells for 2 h. Non-specific binding was determined in the presence of 20 μM of α-cobratoxin (1 h pre-incubation). The filtration on GF/C filters was performed as mentioned above.

The equilibrium binding data were fitted using ORIGIN 7.5 to a one-site model according to the Equation: B(x) = B_max_/(1 + K_d_/x), where B(x) is the radioligand specifically bound at free concentration x (determined by subtracting the amount of bound and adsorbed radioligand from the total amount added to the incubation mixture), B_max_ is the maximal specific bound radioligand, and K_d_ is the dissociation constant.

To evaluate the dissociation kinetics of the [^125^I]-labeled PnIA[R9, L10] from the α7 nAChR, the binding of the 0.4 nM radioligand was allowed to reach equilibrium (2 h incubation), after which a saturating concentration of the α-cobratoxin (20 μM) was added to prevent any re-association of the radioligand to the receptor. The reaction was terminated by rapid filtration on the GF/C filters as mentioned above for different time intervals (2 to 120 min).

### Analysis of a-Conotoxin PnIA Analog Interaction with the a_7_-nAChR via Two-Electrode Voltage Clamp Electrophysiology

Oocytes were prepared from mature female *Xenopus laevis* by following the standard procedure described elsewhere^50^. After a rat α7 nAChR cDNA injection, the oocytes were incubated at 18 °C for 48–72 hours and analyzed via electrophysiology measurements. The membrane potential was clamped at −70 mV using a turbo TEC-03X amplifier (npi electronic, Germany). The data were collected and handled using either Patch Master or WinWCP software. Acetylcholine applications (20 s) were performed every 5 minutes. To test activity, the conotoxin analogs were applied 4 minutes before acetylcholine application. The amplitudes of acetylcholine-evoked currents were measured and normalized to control amplitudes of the acetylcholine response.

### Analysis of a-Conotoxin PnIA Analog Interaction with Muscle-Type nAChR via Calcium Imaging

The activity of the α-conotoxin PnIA[R5, R9, L10, R14] analog towards the muscle-type nAChR was examined on the mouse α_1_β_1_δε-nAChR, heterologously expressed in the neuroblastoma Neuro2a cell line, where an acetylcholine-induced increase in intracellular Ca^2+^ concentration ([Ca^2+^]_i_) is registered. The co-expression of Case12, a fluorescent genetically encoded sensor of calcium ions, allowed us to directly monitor the changes in [Ca^2+^]_i_. Mouse neuroblastoma Neuro2a cells grown in a black 96-well plate in DMEM, supplemented with 10% fetal bovine serum (GE Healthcare HyClone, USA), were transiently transfected with plasmids coding the mouse α_1_β_1_δε-nAChR (mouse α_1_-, β_1_-, δ-, and ε-nAChR-pRBG4), and a fluorescent calcium sensor Case12 (pCase12-cyto vector, Evrogen, Russia) following a lipofectamine transfection protocol (Invitrogen, USA). Intracellular calcium concentration [Ca^2+^]_i_ measurements were performed in an external buffer containing 140 mM NaCl, 2 mM CaCl_2_, 2.8 mM KCl, 4 mM MgCl_2_, 20 mM HEPES, 10 mM glucose, pH 7.4 at 25 °C. The cells were incubated with the PnIA[R5, R9, L10, R14] analog (0.55 μM) for 20 min at room temperature before adding acetylcholine iodide (Sigma, Germany). Changes in the fluorescence of the calcium sensor Case12 (ex/em = 491/516 nm) were detected with the microplate reader HidexSence (Hidex, Turku, Finland) every 2 s for three minutes. The responses were measured as peak intensity minus basal fluorescence level. The data files were analyzed using HidexSence software (Hidex, Turku, Finland) and OriginPro 7.5 software (OriginLab, MA, USA).

## Additional Information

**How to cite this article**: Kasheverov, I. E. *et al*. High-Affinity α-Conotoxin PnIA Analogs Designed on the Basis of the Protein Surface Topography Method. *Sci. Rep*. **6**, 36848; doi: 10.1038/srep36848 (2016).

**Publisher’s note:** Springer Nature remains neutral with regard to jurisdictional claims in published maps and institutional affiliations.

## Supplementary Material

Supplementary Information

## Figures and Tables

**Table 1:** Activity of α-conotoxin PnIA analogs tested in competition binding assays.

α-Conotoxin PnIA analogs	Binding parameters — IC_50_ in nM (Hill coefficient)				
	in competition with [^125^I]-labeled αBgt for…			in electrophysiology for…	in competition with [^125^I]-labeled PnIA[R9, L10] for…
	*Ls*-AChBP	*Ac*-AChBP	α7 nAChR	α7 nAChR	α7 nAChR
PnIA[R9]	58 ± 7(1.1 ± 0.1)	41 ± 5(1.1 ± 0.1)	2400 ± 200(0.86 ± 0.03)	27 ± 10	1490 ± 280 (1.3 ± 0.3)
PnIA[R9, L10]	18 ± 3(0.86 ± 0.10)	47 ± 5(0.86 ± 0.06)	270 ± 10(0.87 ± 0.03)	27 ± 11	15 ± 6 (high affinity)~1000 (low affinity)
PnIA[R5, R9, L10, R14]	1.22 ± 0.04(2.4 ± 0.2)	24 ± 2(0.92 ± 0.06)	860 ± 20(1.4 ± 0.1)	17 ± 2	390 ± 75 (1.8 ± 0.6)

All the listed peptides were evaluated for their ability to compete with [^125^I]-labeled αBgt in binding to AChBPs and α7 nAChR.

**Table 2:** Structures of α-conotoxin PnIA analogs.

α-Conotoxin PnIA analogs	Amino acid sequences	Molecular weight (*МН*^*+*^, Da)	
		calculated	measured
PnIA	GCCSLPPCAANNPDYC*	1623.8	—
PnIA[R9]	GCCSLPPCRANNPDYC*	1708.9	1708.1
PnIA[R9, L10]	GCCSLPPCRLNNPDYC*	1751.0	1750.2
PnIA[R5, R9, L10, R14]	GCCSRPPCRLNNPRYC*	1835.1	1834.3

The asterisk (*) shows the amidated C terminus. As in the natural α-conotoxin PnIA, disulfide bonds in all peptides were formed between Cys2–Cys8 and Cys3–Cys16 residues using orthogonal protecting groups. The substitutions introduced in the analogs are marked underlined.
