# Supplementary material for: High-Affinity α-Conotoxin PnIA Analogs Designed on the Basis of the Protein Surface Topography Method
Source: Sci Rep. 2016 Nov 14;6:36848. doi: 10.1038/srep36848 (PMC5107951; doi:10.1038/srep36848)
Supplement: Supplementary Information [file srep36848-s1.pdf]

Supplementary information

High-Affinity α-Conotoxin PnIA Analogs Designed on the Basis of Protein Surface Topography Method

Igor E. Kasheverov[1*], Anton O. Chugunov[1*], Denis S. Kudryavtsev[1], Igor A. Ivanov[1], Maxim N. Zhmak[1], Irina V. Shelukhina[1], Ekaterina N. Spirova[1], Valentin M. Tabakmakher[2,3], Elena A. Zelepuga[3], Roman G. Efremov[1,4] and Victor I. Tsetlin[1#]

[1] From the Shemyakin-Ovchinnikov Institute of Bioorganic Chemistry, Russian Academy of Sciences,16/10 Miklukho-Maklaya Street, 117997 Moscow, Russia;

[2] From the School of Biomedicine, Far Eastern Federal University, Bldg. M, 10, Ajax Village, Russky Island,Vladivostok 690000, Russia;

[3] From the G.B. Elyakov Pacific Institute of Bioorganic Chemistry, Far Eastern Branch, Russian Academy of Sciences, 159, Prospect 100 let Vladivostoku, 690022 Vladivostok, Russia.

[4] From the National Research University Higher School of Economics, Myasnitskaya ul.20, 101000 Moscow, Russia.

* These authors contributed equally to this work.

# To whom correspondence should be addressed: Victor I. Tsetlin, Shemyakin-Ovchinnikov Institute of Bioorganic Chemistry, Russian Academy of Sciences, 16/10 Miklukho-Maklaya str., 117997 Moscow, Russia. Tel.: 7-495-335 57 33; E-mail: victortsetlin3f@gmail.com.

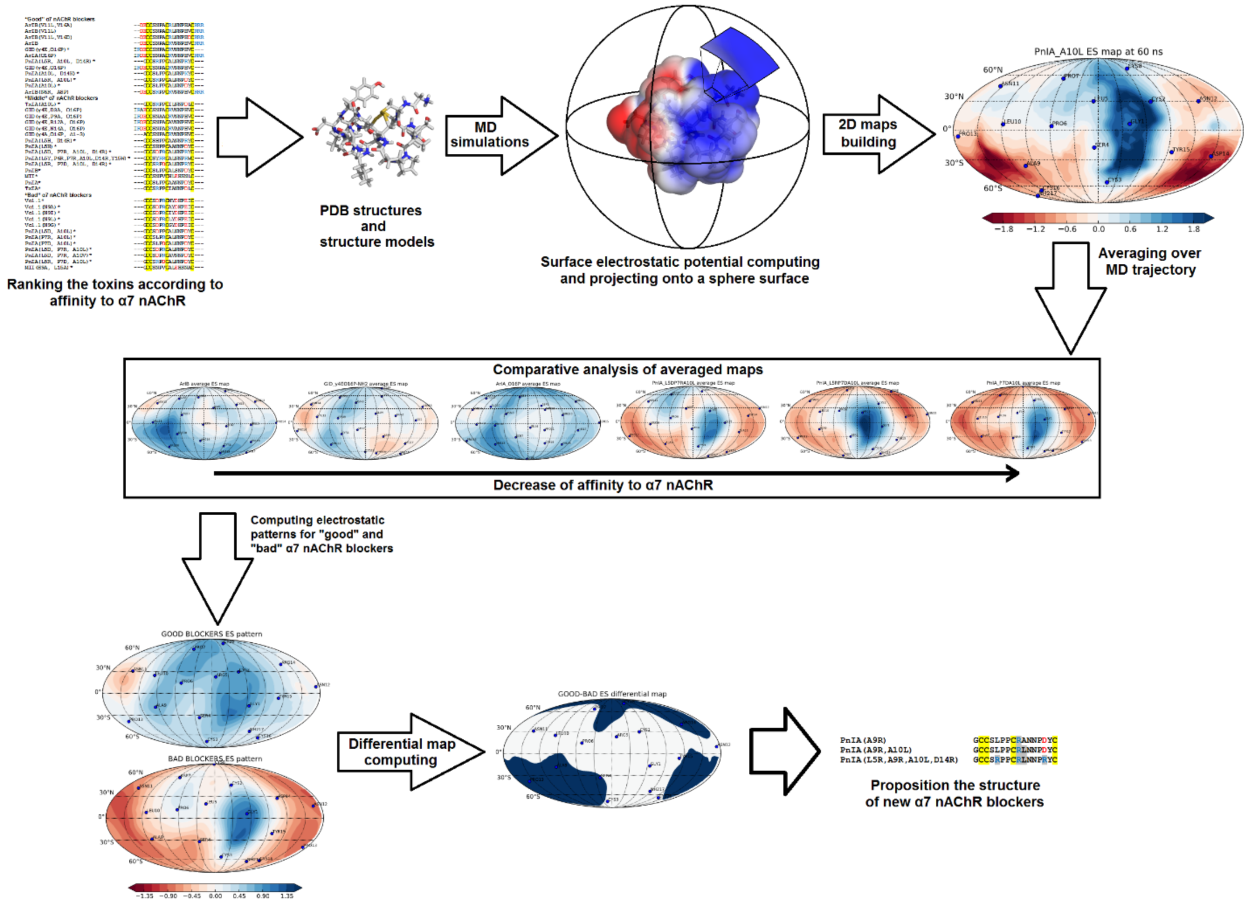

Figure S1. Flowchart of the computational strategy to design high-affinity α7 nAChR blocker based on PnIA conotoxin framework. (A) Establishment of database of α7 nAChR-active conotoxins and their mutants, and stratification according to the channel activity (IC_{50}/K_d). (B) Acquisition of 3D structures from PDB and homology modeling. (C) Molecular dynamics (MD) simulations to account molecular flexibility. (D) Calculation of distribution of conotoxins' electrostatic potential (ELP) on the molecular surface. (E) Building of ELP 2D projection maps with use of Protein Surface Topography (PST) approach. (F) MD-averaging of these maps. (G) Comparative analysis of ELP maps with respect to conotoxins' activity. (H) Computing of characteristic patterns for "good" and "bad" α7 nAChR blockers (group averaging of ELP maps). (I) Building differential map to guide the design of point mutations to improve activity. (J) Three PnIA mutant variants that were synthesized and biochemically tested. Spherical maps for this figure were prepared with our in-house Protein Surface Topography software[1], which is currently available only on request.

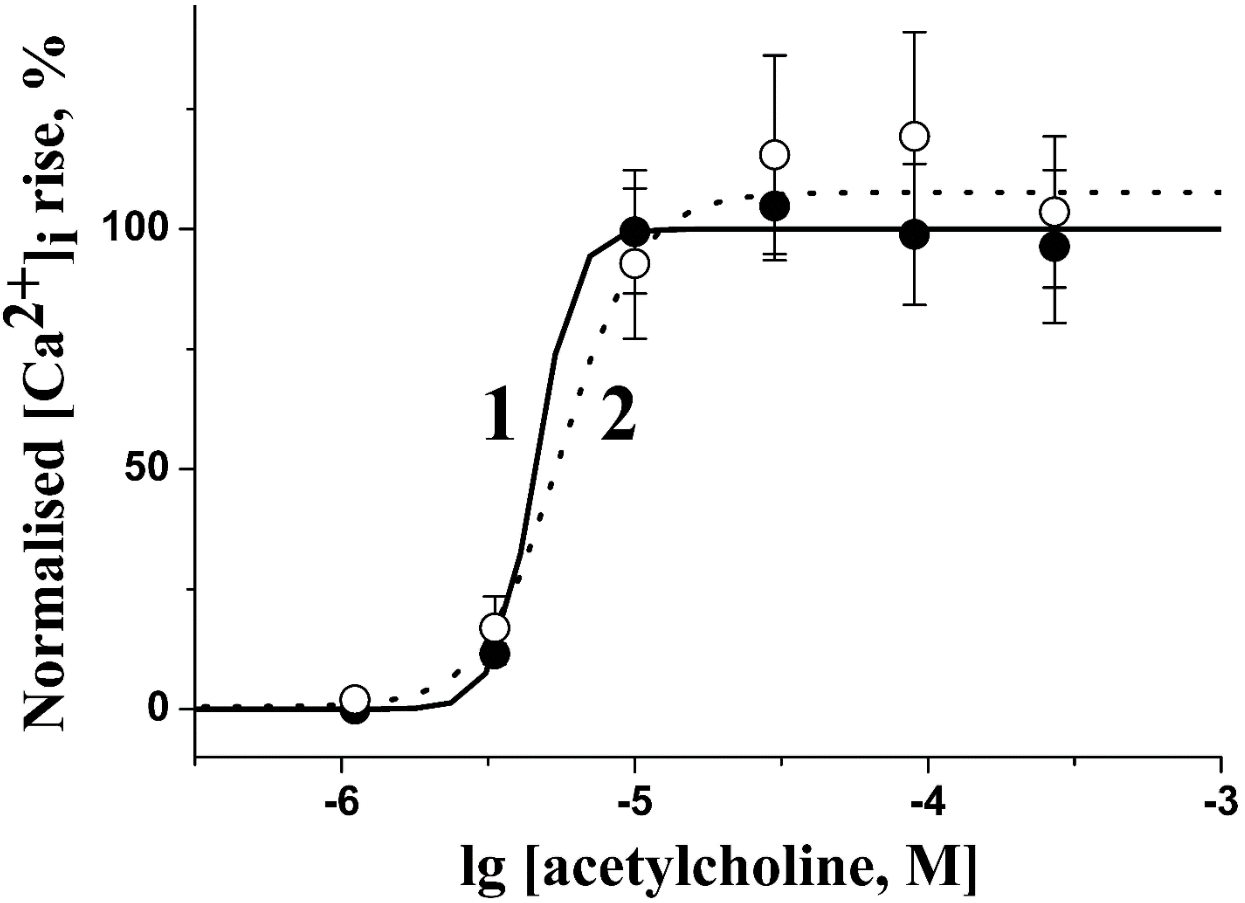

Figure S2. $[Ca^{2+}]_i$ rise in neuroblastoma Neuro2a cells transiently expressing mouse α1β1δε nAChR in response to different concentration of acetylcholine measured in the absence (1, solid line and filled circles) or in the presence of 0.55 µM PnIA[R5, R9, L10, R14] (2, dotted line and open circles).

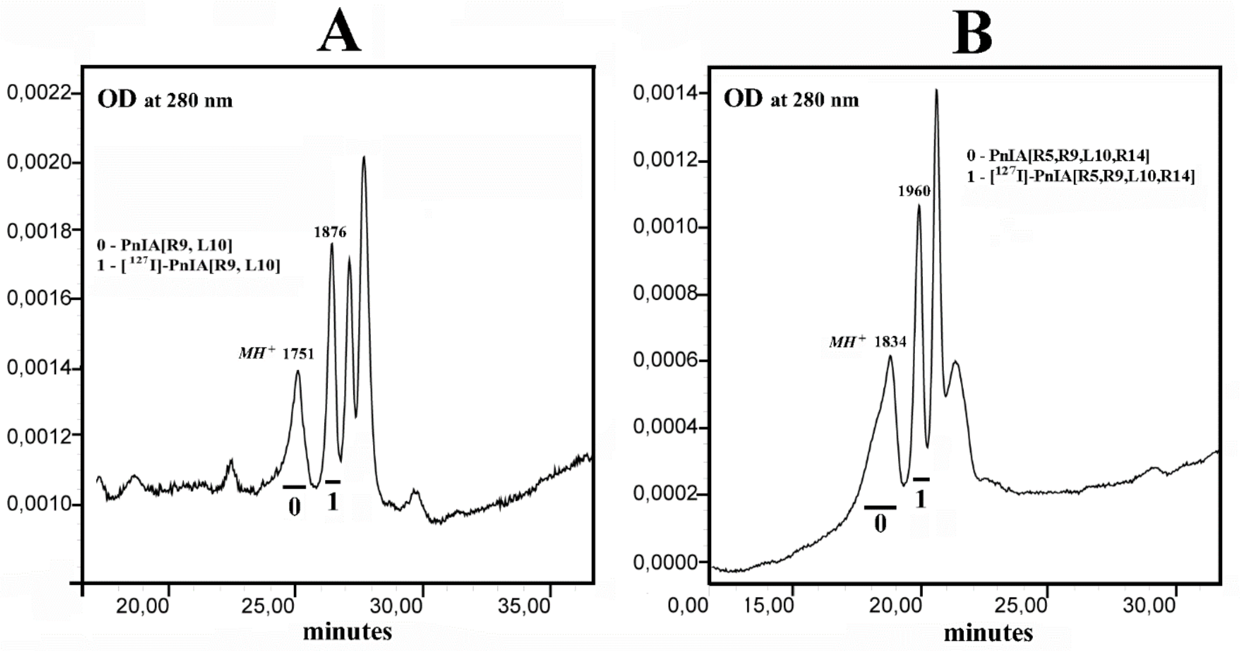

Figure S3. HPLC profiles for the products of the [^{127}I]-iodination reaction of PnIA[R9, L10] (**A**) and PnIA[R5, R9, L10, R14] (**B**). The collected and analyzed peaks of non-modified analogs (**0**) and respective mono-iodinated derivatives (**1**) are marked with indicated molecular masses (*MH*$^+$) measured by MALDI mass-spectrometry.

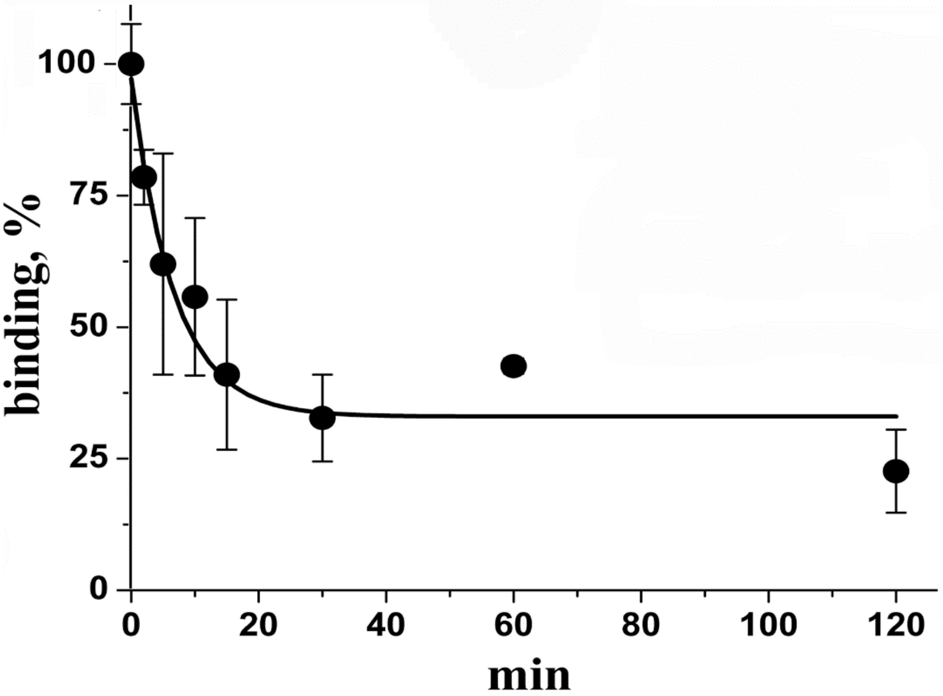

Figure S4. Kinetics of [^{125}I]-PnIA[R9, L10] washout from α7 nAChR transfected in GH_4C_1 cells. Binding of 0.4 nM radioligand was allowed to reach equilibrium (2 h incubation) followed by adding of α-cobratoxin (20 µM) at the indicated time (from 2 min till 2 h). Each point is a mean ± s.e.m. value of two measurements for each time interval in single experiments.

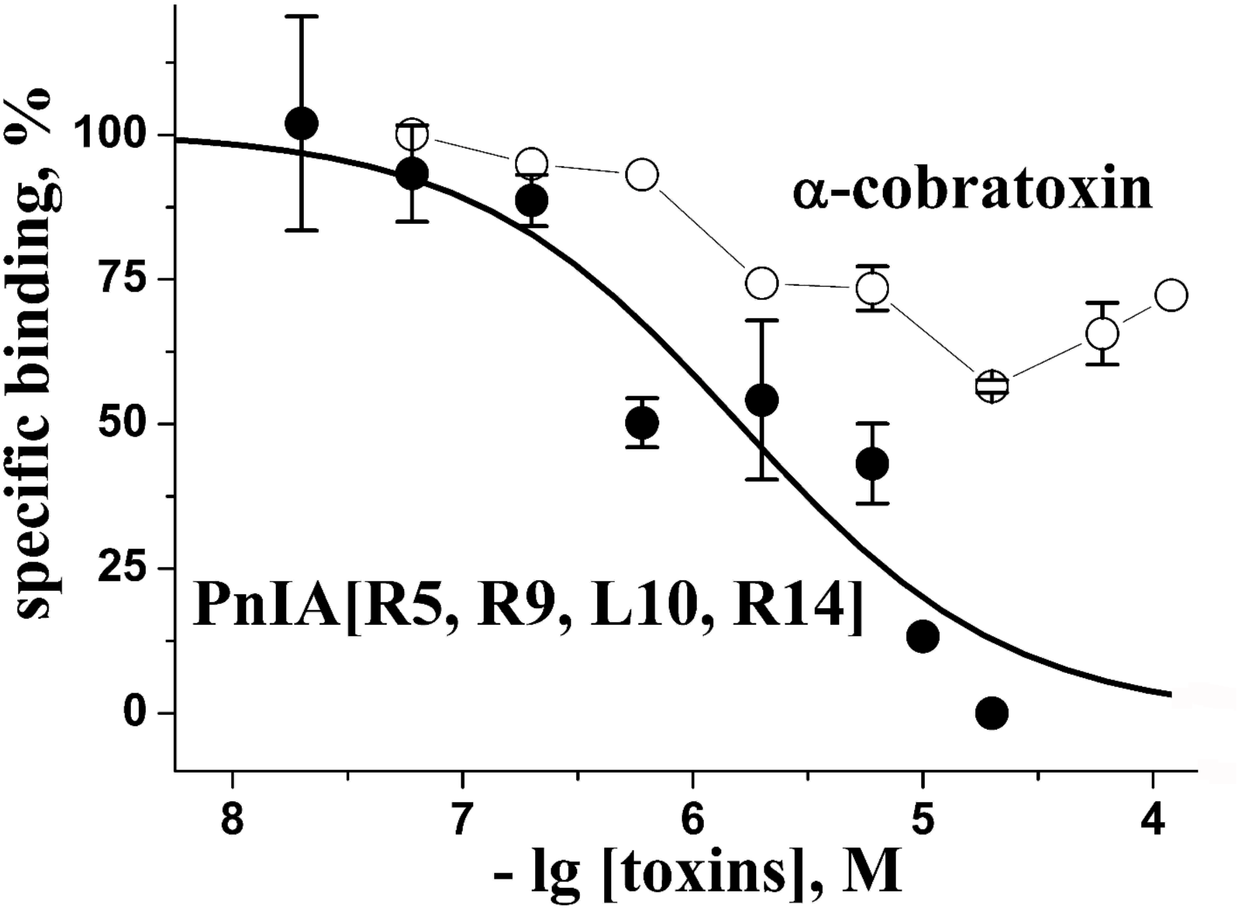

Figure S5. Inhibition of [^{125}I]-PnIA[R5, R9, L10, R14] binding to α7 nAChR transfected in GH_4C_1 cells by α-cobratoxin (open circles, thin line) and α-conotoxin PnIA[R5, R9, L10, R14] analog (filled circles, thick line) with the IC_{50} = 1.6 ± 0.4 μM for the latter (mean ± s.e.m.). Each point is a mean ± s.e.m. value of two measurements for each concentration in two independent experiments. The curve for PnIA[R5, R9, L10, R14] analog was calculated from the means ± s.e.m. using the ORIGIN 7.5 program (see *Methods*).

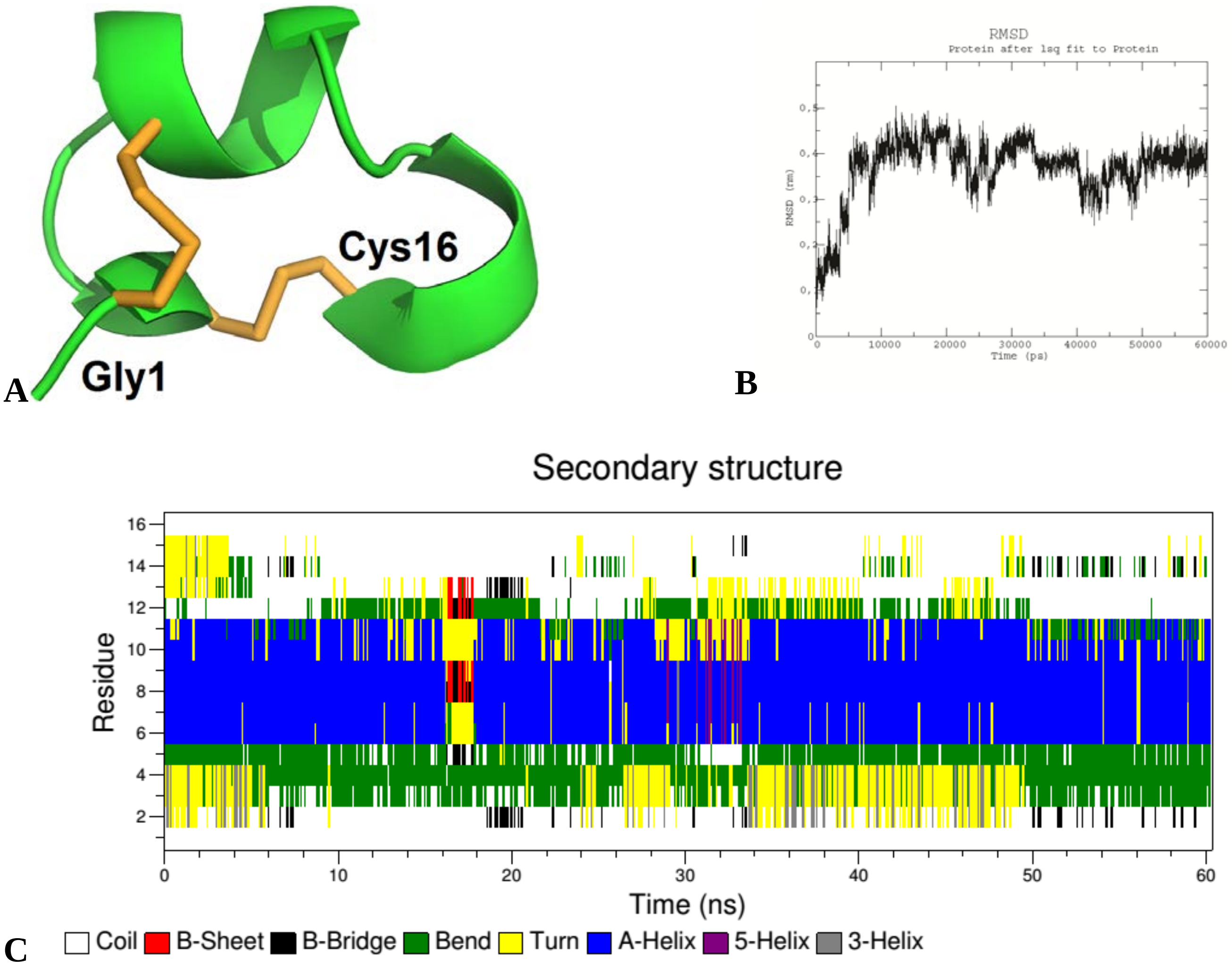

Figure S6. Stability of conotoxin variant PnIA [A10L, D14K] in molecular dynamics (MD) simulations. (A) 3D structure of this conotoxin (PDB ID: 2BR8). Secondary structure is shown as flat ribbons. Disulfide bridges are shown as brown sticks. (B) Root mean square deviation (RMSD) from the starting structure in MD trajectory for this peptide. (C) Secondary structure of this peptide during MD.

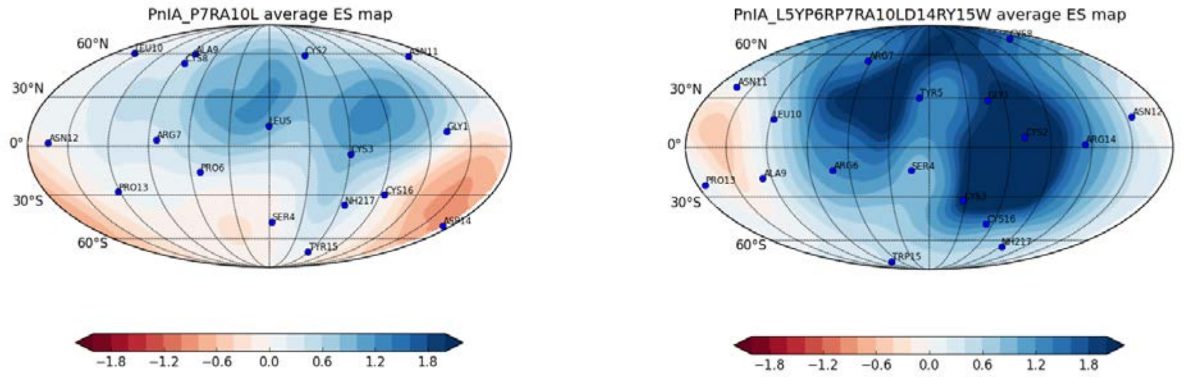

Figure S7. Maps of the electrostatic potential for two PnIA variants (PnIA[P7R, A10L]; *left*, and PnIA[L5Y,P6R,P7R,A10L,D14R,Y15W]; *right*) that possess overall positive net charge and exhibit areas of positive electrostatic potential, but moderately or low active due to improper distribution of the potential (compare with Fig. 1A and D in the main text of the article).

Table S1. Three groups of α-conotoxins and their mutants according to α7 nAChR activity. "Good", IC50<16 nM; "average", 39 nM< IC50 < 390 nM; and "bad", IC50 > 390 nM.

Toxin name	Sequence[$]<br>1 5 9 16	α7 nAChR activity<br>(IC_{50}, nM)	Ref
	Good		
ArIB (V11L, V16A)	DECCSNPACRLNNPHACRRR	0.356[a]	2
ArIB (V11L)	DECCSNPACRLNNPHVCRRR	0.539[a]	2
ArIB (V11L, V16D)	DECCSNPACRLNNPHDCRRR	1.09[a]	2
ArIB	DECCSNPACRVNNPHVCRRR	1.81[a]	2
GID (γ4E, O16P)[#]	IRDECCSNPACRVNNPHVC-NH_2	2.0 ± 0.1[a]	3
ArIA(O16P)[#]	IRDECCSNPACRVNNPHVCRRR	6.02[a]	2
GID (γ4E, O16P)[#]	IRDECCSNPACRVNNPHVC	8.4 ± 1.9[a]	3
PnIA (L5R, A10L, D14R)	GCCSRPPCALNNPRYC-NH_2	10[a]; 670 ± 50[b]	4, 5
PnIA (A10L, D14K)	GCCSLPPCALNNPKYC-NH_2	7200 ± 700[b]	5
PnIA (L5R, A10L)	GCCSRPPCALNNPDYC-NH_2	12000 ± 2000[b]	5
PnIA (A10L)	GCCSLPPCALNNPDYC-NH_2	12.6[a]; 14000 ± 1000[b]	2, 5
ArIB (N6R, A8P)	DECCSRPPCRVNNPHVCRRR	15.9[a]	2
	Average		
TxIA (A10L)	GCCSRPPCILNNPDLC-NH_2	39[a]	4
GID(D3A, γ4E, O16P)[#]	IRAECCSNPACRVNNPHVC	42.1 ± 10.9[a]	3
GID(γ4E, P9A, O16P)[#]	IRDECCSNAACRVNNPHVC	90.5 ± 22.6[a]	3
GID(γ4E, R12A, O16P)[#]	IRDECCSNPACAVNNPHVC	48.8 ± 4.2[a]	3
GID(γ4E, N14A, O16P)[#]	IRDECCSNPACRVANPHVC	51.4 ± 6.8[a]	3
GID (Δ1–3, γ4A, O16P)[#]	ACCSNPACRVNNPHVC	100.3 ± 8.3[a]	3
PnIA (L5H, D14R)	GCCSHPPCAANNPRYC-NH_2	21000 ± 1000[b]	5
PnIA (L5H)	GCCSHPPCAANNPDYC-NH_2	26000 ± 1000[b]	5
PnIA (L5D, P7R, A10L, D14R)	GCCSDPRCALNNPRYC-NH_2	23000 ± 1000[b]	5
PnIA (L5Y,P6R,P7R,A10L,D14R,Y15W)	GCCSYRRCALNNPRWC-NH_2	19000 ± 1000[b]	5
PnIA (L5R, P7D, A10L, D14R)	GCCSRPDCALNNPRYC-NH_2	72000 ± 5000[b]	5
PnIB	GCCSLPPCALSNPDYC-NH_2	61.3[b]	6
MII	GCCSNPVCHLEHSNLC-NH_2	130[a]	7
PnIA	GCCSLPPCAANNPDYC-NH_2	252[a]	2
TxIA	GCCSRPPCIANNPDLC-NH_2	390[a]	4
	Bad		
Vc1.1	GCCSDPRCNYDHPEIC-NH_2	7123[a]	8
Vc1.1(N9A)	GCCSDPRCAYDHPEIC-NH_2	>3000[a]	8
Vc1.1(N9I)	GCCSDPRCIYDHPEIC-NH_2	963[a]	8
Vc1.1(N9L)	GCCSDPRCLYDHPEIC-NH_2	>3000[a]	8
Vc1.1(N9G)	GCCSDPRCGYDHPEIC-NH_2	>3000[a]	8
PnIA (L5D, A10L)	GCCSDPPCALNNPDYC-NH_2	>100000[b]	5
PnIA (P7R, A10L)	GCCSLPRCALNNPDYC-NH_2	>100000[b]	5
PnIA (P7D, A10L)	GCCSLPDCALNNPDYC-NH_2	>100000[b]	5
PnIA (L5D, P7R, A10L)	GCCSDPRCALNNPDYC-NH_2	>100000[b]	5
PnIA (L5D, P7R, A10V)	GCCSDPRCAVNNPDYC-NH_2	>100000[b]	5
PnIA (L5R, P7D, A10L)	GCCSRPDCALNNPDYC-NH_2	>100000[b]	5
MII (H9A, L15A)	GCCSNPVCALEHSNAC-NH_2	>10000[a]	9

[$] — Sequences are aligned, and positions are numbered according to PnIA.

[#] — O=oxyproline. Although, in this work proline analog was used for computations, since according to [3] this substitution almost does not affectactivity.

[a] — IC_{50} (nM) for blocking of *Xenopus* oocyte-expressed α7 nAChR; [b] — IC_{50} (nM) in [^{125}I]-αBgt displacement from α7 nAChR.

References

1. Koromyslova, A.D., Chugunov, A.O. & Efremov, R.G. Deciphering fine molecular details of proteins' structure and function with a Protein Surface Topography (PST) method. *J Chem Inf Model* **54**, 1189-99 (2014).
2. Whiteaker, P. et al. Discovery, synthesis, and structure activity of a highly selective alpha7 nicotinic acetylcholine receptor antagonist. *Biochemistry* **46**, 6628-38 (2007).
3. Millard, E.L. et al. Inhibition of neuronal nicotinic acetylcholine receptor subtypes by alpha-Conotoxin GID and analogues. *J Biol Chem* **284**, 4944-51 (2009).
4. Dutertre, S. et al. AChBP-targeted alpha-conotoxin correlates distinct binding orientations with nAChR subtype selectivity. *EMBO J* **26**, 3858-67 (2007).
5. Kasheverov, I.E., Zhmak, M.N., Khruschov, A.Y. & Tsetlin, V.I. Design of new alpha-conotoxins: from computer modeling to synthesis of potent cholinergic compounds. *Mar Drugs* **9**, 1698-714 (2011).
6. Luo, S. et al. Single-residue alteration in alpha-conotoxin PnIA switches its nAChR subtype selectivity. *Biochemistry* **38**, 14542-8 (1999).
7. Lindstrom, J.M. Nicotinic acetylcholine receptors of muscles and nerves: comparison of their structures, functional roles, and vulnerability to pathology. *Ann N Y Acad Sci* **998**, 41-52 (2003).
8. Halai, R. et al. Scanning mutagenesis of alpha-conotoxin Vc1.1 reveals residues crucial for activity at the alpha9alpha10 nicotinic acetylcholine receptor. *J Biol Chem* **284**, 20275-84 (2009).
9. McIntosh, J.M. et al. Analogs of alpha-conotoxin MII are selective for alpha6-containing nicotinic acetylcholine receptors. *Mol Pharmacol* **65**, 944-52 (2004).
